# Evaluating predictors of kinase activity of STK11 variants identified in primary human non-small cell lung cancers

**DOI:** 10.1007/s00439-025-02726-0

**Published:** 2025-02-12

**Authors:** Yile Chen, Kyoungyeul Lee, Junwoo Woo, Dong-wook Kim, Changwon Keum, Giulia Babbi, Rita Casadio, Pier Luigi Martelli, Castrense Savojardo, Matteo Manfredi, Yang Shen, Yuanfei Sun, Panagiotis Katsonis, Olivier Lichtarge, Vikas Pejaver, David J. Seward, Akash Kamandula, Constantina Bakolitsa, Steven E. Brenner, Predrag Radivojac, Anne O’Donnell-Luria, Sean D. Mooney, Shantanu Jain

**Affiliations:** 1 Department of Biomedical Informatics and Medical Education, University of Washington, Seattle, WA 98105 USA; 2 3billion, 3billion Biotechnology Company, Seoul, South Korea; 3 Department of Pharmacy and Biotechnology, University of Bologna, 40126 Bologna, Italy; 4 Department of Electrical and Computer Engineering, Texas A&M University, College Station, TX 77843 USA; 5 Molecular and Human Genetics, Baylor College of Medicine, Houston, TX 77030 USA; 6 Institute for Genomic Health, Icahn School of Medicine at Mount Sinai, New York, NY 10029 USA; 7 Department of Genetics and Genomic Sciences, Icahn School of Medicine at Mount Sinai, New York, NY 10029 USA; 8 Department of Pathology, University of Vermont, Burlington, VT 5445 USA; 9 Khoury College of Computer Sciences, Northeastern University, Boston, MA 02115 USA; 10 University of California, Berkeley, Berkeley, CA 94720 USA; 11 Division of Genetics and Genomics, Boston Children’s Hospital, Harvard Medical School, Boston, MA 02115 USA; 12 Broad Center for Mendelian Genomics, Program in Medical and Population Genetics, Broad Institute of Massachusetts Institute of Technology and Harvard, Cambridge, MA 02142 USA; 13 Center for Information Technology, National Institutes of Health, Bethesda, MD 20892 USA; 14 The Institute for Experiential AI, Northeastern University, Boston, MA 02115 USA

## Abstract

Critical evaluation of computational tools for predicting variant effects is important considering their increased use in disease diagnosis and driving molecular discoveries. In the sixth edition of the Critical Assessment of Genome Interpretation (CAGI) challenge, a dataset of 28 STK11 rare variants (27 missense, 1 single amino acid deletion), identified in primary non-small cell lung cancer biopsies, was experimentally assayed to characterize computational methods from four participating teams and five publicly available tools. Predictors demonstrated a high level of performance on key evaluation metrics, measuring correlation with the assay outputs and separating loss-of-function (LoF) variants from wildtype-like (WT-like) variants. The best participant model, 3Cnet, performed competitively with well-known tools. Unique to this challenge was that the functional data was generated with both biological and technical replicates, thus allowing the assessors to realistically establish maximum predictive performance based on experimental variability. Three out of the five publicly available tools and 3Cnet approached the performance of the assay replicates in separating LoF variants from WT-like variants. Surprisingly, REVEL, an often-used model, achieved a comparable correlation with the real-valued assay output as that seen for the experimental replicates. Performing variant interpretation by combining the new functional evidence with computational and population data evidence led to 16 new variants receiving a clinically actionable classification of likely pathogenic (LP) or likely benign (LB). Overall, the STK11 challenge highlights the utility of variant effect predictors in biomedical sciences and provides encouraging results for driving research in the field of computational genome interpretation.

**Supplementary Information:**

The online version contains supplementary material available at 10.1007/s00439-025-02726-0.

## Introduction

The STK11 gene, formerly known as LKB1 (Liver Kinase B1), encodes the enzyme Serine/Threonine Kinase 11 (NP_000446.1) that is considered to be a “master kinase” and functions as a tumor suppressor. It regulates many intracellular signaling networks, impacting metabolism, proliferation, transcription, and cell morphology (Hezel and Bardeesy 2008; Lenahan et al. 2024). Unlike most mammalian kinases that are activated by autophosphorylation of their activation loop, STK11 activity is regulated by its interaction with pseudokinase STRAD$\alpha$ and the scaffolding protein MO25 forming a heterotrimeric complex, where its activation loop is stabilized in a conformation competent for substrate binding (Zeqiraj et al. 2009). Autophosphorylation of STK11 occurs outside the activation loop in the kinase (residues 49–309) and C-terminal regulatory (residues 309–433) domains (Sapkota et al. 2002; Baas et al. 2003). The connection between autophosphorylation and the activation of STK11 is still not well understood.

STK11 phosphorylates many members of the microtubule affinity-regulating kinases family, with AMPK being studied most extensively (Lizcano et al. 2004; Nguyen et al. 2013). STK11 plays a significant role in the p53 signaling axis, activated in response to various cellular stresses, such as oncogene activation, DNA damage, and replication stress (Borrero and El-Deiry 2021). It physically associates with p53 in the nucleus and enhances p53’s transcriptional activity, impacting cell proliferation and apoptosis (Zeng and Berger 2006).

The exact mechanism(s) underlying STK11-mediated activation of p53 are still unclear. It is possible that this activation occurs directly through STK11-mediated phosphorylation of p53, or indirectly through the activation of AMPK and NUAK1 (Hou et al. 2011; Zeng and Berger 2006; Donnelly et al. 2021). However, regardless of the mechanism, intact STK11 function is important for p53 activation.

STK11 is a significant disease gene due to its involvement in both the rare genetic disorder, Peutz–Jeghers syndrome (PJS), and cancer. Germline mutations in STK11 lead to uncontrolled cell growth and the formation of polyps in the gastrointestinal tract, characterizing PJS (Zyla et al. 2021; Khanabadi et al. 2023). Somatic alterations in STK11 are most prevalent in lung cancer, however, they are also observed in other cancer types such as breast, head, and neck cancers (Pons-Tostivint et al. 2021; Krishnamurthy et al. 2021). Notably, STK11 variants are frequently observed in non-small cell lung cancer (NSCLC) adenocarcinomas and are associated with poor survival (La Fleur et al. 2019). Recent studies have highlighted the substantial impact of STK11 mutations in the highly prevalent KRAS-driven NSCLC adenocarcinomas, presenting distinct biological characteristics, therapeutic susceptibilities, and immune profiles (Skoulidis et al. 2018). STK11 alterations in KRAS-driven NSCLC adenocarcinomas are associated with low PD-L1 (Programmed Death-Ligand 1) levels, leading to reduced efficacy of anti-PD-1 monoclonal antibody therapy.

Functional and computational characterization of variants in disease genes such as STK11 is critical for the success of genomic medicine (Rost et al. 2016; Shendure et al. 2019; The Critical Assessment of Genome Interpretation Consortium 2024). The increasing rate of genetic testing has resulted in a growing number of newly identified variants. However, the pace of variant discovery has surpassed the rate of variant interpretation. The pathogenicity/benignity of many variants cannot be established conclusively, leading to the *variant of uncertain significance* (VUS) categorization being the largest category in clinical databases (Landrum et al. 2016). Functional assays and computational tools are often used to provide evidence for moving VUS to pathogenic/benign categories and improving variant interpretation (Richards et al. 2015). However, experimentally characterizing the impact of all variants in a disease gene is often infeasible due to costs and technological limitations. Consequently, for many disease genes, only a few variants are characterized functionally. In contrast, computational predictions for pathogenicity and functional effect are readily available for most variants (Zhu et al. 2020), making them a versatile tool for improving variant interpretation, broad functional characterization of underlying mechanisms, and prioritization of experimental studies (Mort et al. 2010; Katsonis et al. 2022; Chen et al. 2023). Thus, continual improvement of computational approaches and their independent evaluation is important.

**Fig. 1:**
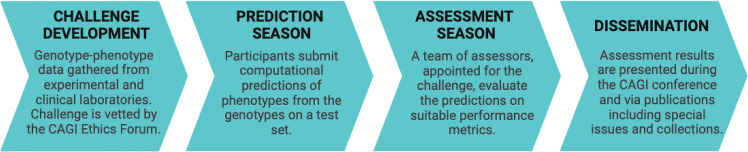
Four stages of a CAGI challenge: All data containing the genotypes or variants and the corresponding phenotypic labels are collected from experimental and clinical labs (data providers) during the Challenge development stage. The prediction season starts when the challenge is opened to the community, inviting computational groups to submit their predictions. If the challenge involves providing a training set, a set of genotypes or variants are released with the ground truth phenotypic labels as the training set. Note that for the CAGI 6 STK11 challenge a training set was not provided. The remaining genotypes or variants are provided as a test set without the ground truth labels. During the prediction season the participants submit phenotype predictions on the test set. The ground truth phenotypic labels are typically made public by the data providers after the prediction season, provided the data does not contain any sensitive information. Additionally the data is also made available on the CAGI website, accessible to researchers after signing a data use agreement. During the assessment season, assessors are appointed to evaluate the performance of the submitted predictions and state of the art baseline methods on the test set. The identity of the participating teams are not revealed to assessors until they present the assessment results to the larger community via online events organized for this purpose. During the Dissemination stage the results are presented to a wider audience though the CAGI conference and publications under the CAGI special issues or collections

To facilitate a thorough and unbiased evaluation of computational tools, the Critical Assessment for Genome Interpretation (CAGI) consortium has worked with several experimental groups to incorporate functional data from recent studies for a blind assessment of predictors in a number of challenges (The Critical Assessment of Genome Interpretation Consortium 2024). Since the functional data is not available in the public domain during or before the prediction submission window, it cannot be used in model training. The approach ensures that the tools’ performance is characterized accurately, unaffected by model overfitting to training data, thereby also ensuring a fair comparison between tools. Each CAGI challenge is undertaken in four stages: (1) Challenge development, (2) Prediction season, (3) Assessment season and (4) Dissemination (Fig. 1). The STK11 challenge, in the sixth CAGI edition, invited computational groups to submit their predictions on 28 coding variants (all but one missense) found in NSCLC biopsies, that were functionally profiled with an in vitro gel-shift assay measuring autophosphorylation and a cell-based p53-dependent luciferase reporter assay (Donnelly et al. 2021). Four participating models and five publicly available tools were evaluated and compared using the functional data on key evaluation metrics. The experimental replicates were used to quantify the consistency of the assay, to establish an upper limit on the predictive performance due to experimental variability, and to assess whether the predictors are comparable to the assays in characterizing the variants’ kinase activity. Lastly, clinical variant classification was performed by combining the evidence from the functional assays, computational tools, allele frequency from population data and other co-located pathogenic variants to move variants with uncertain significance to clinically actionable categories.

## Challenge design and participation

A total of 28 STK11 (NP_000446.1) variants from primary non-small cell lung cancer (NSCLC) biopsy specimens were assessed for biological impact in Dr. Seward’s laboratory at the Department of Pathology and Laboratory Medicine, University of Vermont. The variants were released to the community through the CAGI website, inviting computational groups to submit their predictions for each variant’s kinase activity. The challenge was publicly announced on May 20, 2021, the set of variants was released on June 8, 2021, and the submissions were accepted from June 21, 2021, to August 31, 2021. A relatively short prediction season was impacted by the timeline for the public release of the ground truth data (Donnelly et al. 2021).

The participants were asked to calibrate their predictions as the percentage of the wildtype activity retained divided by 100, wherein 0 indicates no activity, 1 indicates wildtype activity and values of 0.2 and 1.2 indicate 20% and 120% wildtype activity. The submitted predictions were evaluated against experimentally validated kinase activities. Four teams participated in the challenge, collectively submitting 14 predictors (Tables 1, 2). Two teams submitted six predictors each and the other two teams submitted one predictor each. In addition to evaluating the submitted predictions, we also evaluated five publicly available tools as baselines; see “Models and baselines”.

**Table 1:** Table listing each predictor, its main reference if available, types of features utilized, and sources of training data

Method name	References	PolyPhen, SIFT, Provean based features	Structure based features	PSSM, MSA based features	Model	Database
3Cnet	Won et al. (2021)	No	Yes	Yes	Neural Network, Random Forests	ClinVar, gnomAD, UniRef
Evolutionary action	Katsonis and Lichtarge (2014)	No	No	Yes	Mathematical model for evolutionary action of variants on fitness	UniRef & nr NCBI
Protein language model	Sun and Shen (2023)	No	No	No	BERT-based masked language modeling	Pfam-rp15, UniProt
Bologna biocomputing	Savojardo et al. (2016), Manfredi et al. (2021)	No	INPS3D: Yes; DeepREx: No	Yes	INPS3D: Support Vector Regression; DeepREx: Stack of LSTM layers	INPS3D: S2648; DeepREx: PDB, UniProt

**Table 2:** Table showing each predictor and individuals involved in developing or submitting to the STK11 CAGI challenge

Team	Members
3Cnet	Kyoungyeul Lee
	Junwoo Woo
	Dong-wook Kim
	Changwon Keum
Evolutionary action	Panagiotis Katsonis
	Olivier Lichtarge
Protein language model	Yang Shen
	Yuanfei Sun
Bologna biocomputing	Giulia Babbi
	Rita Casadio
	Pier Luigi Martelli
	Castrense Savojardo
	Matteo Manfredi

## Experimental data

**Fig. 2:**
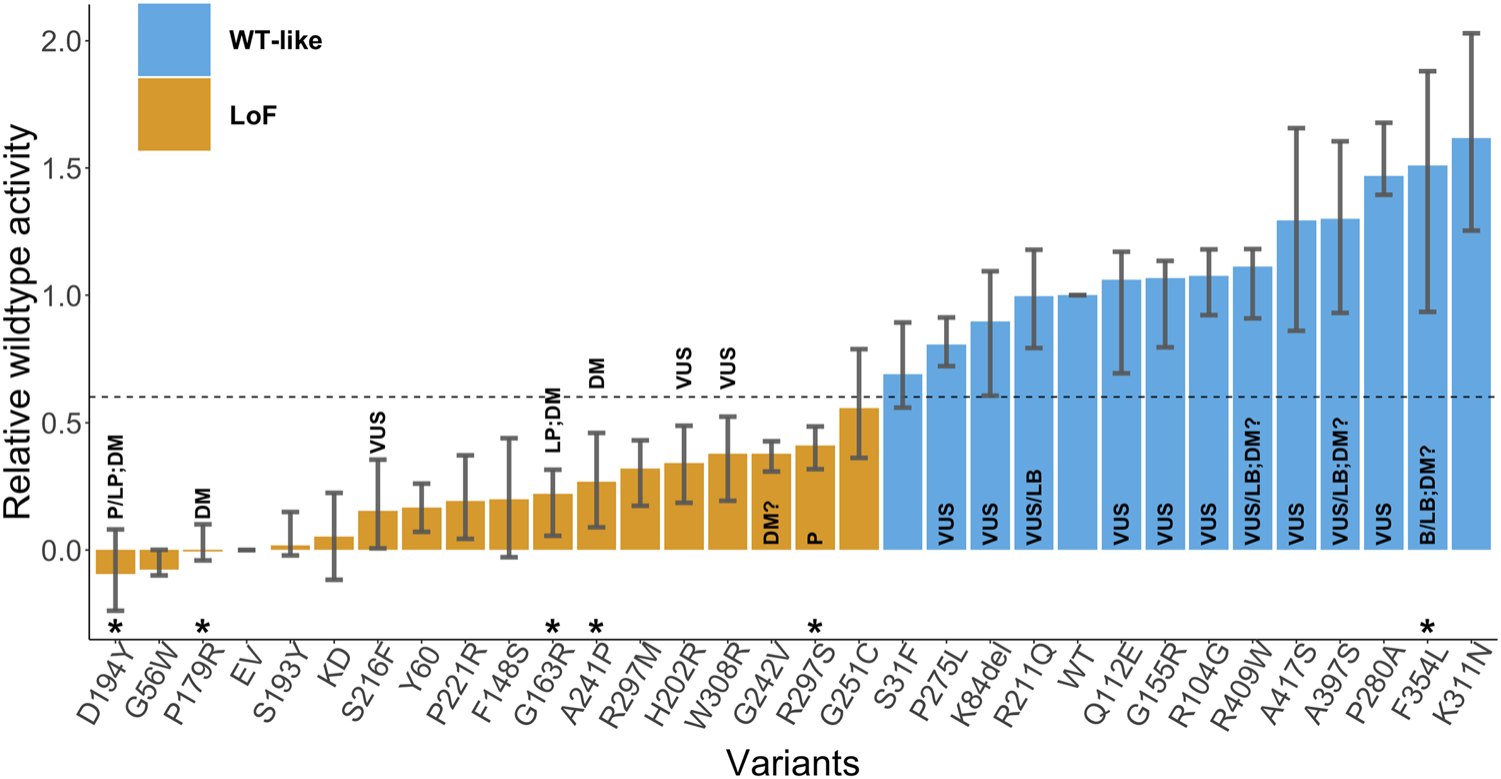
Relative wildtype (R-WT) activity of each variant measured by the luciferase assay. The average activity over the biological and technical replicates is shown along with the 25th and the 75th percentile. LoF variants are displayed in orange, and WT-like variants in blue, separated based on an R-WT activity threshold of 0.6. Any variant with an asterisk above its identifier was classified without conflicts as pathogenic or benign in ClinVar (2024-01-27) (Landrum et al. 2016) and/or a disease mutation in HGMD (2021-04) (Stenson et al. 2020) and has not been used in the assessment. Labels inside or on top of the bars indicate clinical classification in ClinVar and HGMD, including pathogenic (P), likely pathogenic (LP), variant of uncertain significance (VUS), benign (B), likely benign (LB), disease-causing mutation (DM), and possible disease-causing mutation (DM?). Abbreviations EV, KD, and WT stand for empty vector, kinase-dead point mutation (p.K78I), and wildtype, respectively

The CAGI6 STK11 challenge presented 27 missense variants and 1 single amino acid deletion (Fig. 2, Table 3) identified in primary NSCLC biopsy specimens with <1% allele frequency in gnomAD (Karczewski et al. 2020). The STK11 activity of each variant was assessed experimentally via (1) a luciferase reporter assay, measuring an STK11 variant’s effect on TP53’s transcriptional activity, and (2) a gel-shift assay, putatively measuring whether an STK11 variant undergoes auto-phosphorylation or not (Donnelly et al. 2021).

**Table 3:** CAGI6 STK11 challenge dataset of 28 variants found in primary NSCLC biopsy specimens

p.SYNTAX (NP_000446.1)	chr19:g.SYNTAX (GRCh37)	ClinVar	HGMD	gnomAD	gnomAD	R-WT activity [25%, 75%]	TP53 mediated	Autophosphorylation
				AC	AF		Luciferase assay result	Assay result
D194Y*	g.1220487G >T	P/LP	DM			$-$ 0.09 [$-$ 0.24, 0.08]	LoF	LoF
G56W	g.1207078G>T					$-$ 0.08 [$-$ 0.1, 0]	LoF	LoF
P179R*	g.1220443C>G		DM			$-$ 0.01 [$-$ 0.04, 0.1]	LoF	LoF
S193Y	g.1220485C>A					0.02 [$-$ 0.02, 0.15]	LoF	LoF
S216F	g.1220629C>T	VUS				0.15 [0.01, 0.36]	LoF	LoF
P221R	g.1220644C>G					0.19 [0.04, 0.37]	LoF	LoF
F148S	g.1219391T>C					0.20 [$-$ 0.03, 0.44]	LoF	LoF
G163R*	g.1220394G>C	LP	DM			0.22 [0.06, 0.32]	LoF	LoF
A241P*	g.1220703G>C		DM			0.27 [0.09, 0.46]	LoF	LoF
R297M	g.1221975G>T					0.32 [0.17, 0.43]	LoF	LoF
H202R	g.1220587A>G	VUS		5	3.16E−06	0.34 [0.19, 0.49]	LoF	WT
W308R	g.1222985T>C	VUS				0.38 [0.19, 0.52]	LoF	LoF
G242V	g.1220707G>T		DM?			0.38 [0.31, 0.43]	LoF	LoF
R297S*	g.1221976G>T	P				0.41 [0.32, 0.49]	LoF	LoF
G251C	g.1221228G>T					0.56 [0.36,0.79]	LoF	LoF (weak)
S31F	g.1207004C>T					0.69 [0.56, 0.89]	WT	WT
P275L	g.1221301C>T	VUS				0.81 [0.72, 0.91]	WT	WT
K84del	g.1207153_1207155delAAG	VUS		7	4.34E−06	0.90 [0.61, 1.09]	WT	WT
R211Q	g.1220614G>A	VUS/LB		29	1.81E−05	1.00 [0.79, 1.18]	WT	WT
Q112E	g.1218459C>G	VUS		2	1.24E−06	1.06 [0.69, 1.17]	WT	WT
G155R	g.1219411G>A	VUS		3	1.90E−06	1.07 [0.8, 1.13]	WT	WT
R104G	g.1218435A>G	VUS		6	3.72E−06	1.08 [0.92, 1.18]	WT	WT
R409W	g.1226569C>T	VUS/LB	DM?	68	4.28E−05	1.11 [0.91, 1.18]	WT	WT
A417S	g.1226593G>T	VUS		16	1.02E−05	1.29 [0.86, 1.66]	WT	WT
A397S	g.1226533G>T	VUS/LB	DM?	30	1.87E−05	1.30 [0.93, 1.6]	WT	WT
P280A	g.1221315C>G	VUS				1.47 [1.39, 1.68]	WT	WT
F354L*	g.1223125C>G	B/LB	DM?	8225	5.10E−03	1.51 [0.93, 1.88]	WT	WT
K311N	g.1222996G>T					1.62 [1.25, 2.03]	WT	WT

Variants excluded from evaluation are marked by an asterisk in the first column. ClinVar (2024-01-27) and HGMD (2021-04) annotations, before the prediction submission deadline, are shown along with experimental results, as well as each variant’s allele count (AC) and allele frequency (AF) in gnomAD (v4.1.0)

*VUS* variant of uncertain significance, *P* pathogenic, *LP* likely pathogenic, *B* benign, *LB* likely benign, *DM* disease-causing mutation, *DM?* possible disease-causing mutation

For the luciferase assay plasmids containing cDNAs encoding each of the STK11 variants (STK11/eGFP) were transfected into A549 cells along with a plasmid encoding TP53 response element with a firefly luciferase reporter (PG13-luc) and a transfection control plasmid with Renilla reniformis luciferase reporter (pRL-SV40). The luciferase activity, adjusted for transfection efficiency, serves as a measure of an STK11 variant’s effect on TP53’s transcriptional activity. In addition to the somatic variants, the luciferase activity for the wildtype (WT) STK11, a kinase-dead point mutation (p.K78I), and empty vector (EV) were also measured, as a positive control, negative control, and baseline, respectively. Seventeen biological replicates were performed, each measuring the activity for a subset of STK11 variants, across 2–3 technical replicates. The two controls and baseline (WT, p.K78I, and EV) were measured across all biological and technical replicates. Each variant from the set of 28 cancer biopsy variants was validated in 3–6 biological replicates.

For the gel shift assay, mutant proteins were transfected into A549 cells lacking functional STK11. The STK11 heterotrimeric complexes were immunoprecipitated with anti-Flag beads and kinase assays were performed on the immunoprecipitated complexes. The kinase reactions were then subjected to SDS-PAGE electrophoresis and transferred to nitrocellulose membranes, followed by Western Blot analysis with anti-STK11 monoclonal antibody, and detected with anti-mouse-HRP. The evaluated variants either demonstrated (1) a single unmodified band, representing an inability to auto-phosphorylate, or (2) two bands, an unmodified band and a shifted higher molecular weight band, presumably the result of autophosphorylation (although the possibility of phosphorylation by another cell kinase cannot be excluded) indicating the variant behaved as WT. The addition of phosphatase eliminated the second band, confirming it was the product of phosphorylation. The assay was essentially binary, classifying the variant as WT-like or loss of function (LoF).

The luciferase assay gave a continuous activity value for each variant. The data providers classified each variant as either WT-like or LoF by applying a suitable threshold (Donnelly et al. 2021). The class labels from the two assays agreed on 27 out of the 28 variants, with the disagreement on p.H202R, assigned a LoF label as per the luciferase assay and a WT-like label as per the gel-shift assay.

## Assessment methods

In our assessment, we used the data from the luciferase assay for our main results. The results of the gel shift assay are provided in Supplementary File 8. The two assays agree on the classification labels for all variants, except p.H202R (Table 3). Due to the high agreement between the two assays and the availability of the continuous activity values and replicates for the luciferase assay, we deemed it to be better suited and sufficient for the primary assessment. The predictors were evaluated over a regression and classification task to measure their performance on the luciferase assay.

### Ground truth for evaluation

The luciferase activity measured in the assay was normalized relative to the wildtype activity after correcting for the background activity using the following formula.

$$\begin{aligned} \text {R-WT Activity}_{\text {Var}} = \frac{\text {Activity}_{\text {Var}} - \text {Activity}_{\text {EV}}}{\text {Activity}_{\text {WT}} - \text {Activity}_{\text {EV}}}, \end{aligned}$$

where all the raw activity values come from the same biological and technical replication. The normalization scaled the activity values such that values $\le$0 correspond to no activity, values $=$ 1 correspond to WT activity, and values > 1 correspond to greater than WT activity. Note that the data providers used a different normalization approach that scales the relative activity on a larger scale than 0–1. We used a 0–1 scale based on the CAGI challenge guidelines. The relative wildtype activity (R-WT) for variant *i* was averaged across all biological and technical replicates to give a robust measure of its R-WT activity, which we consider the ground truth for the activity prediction task.

To evaluate the methods on a binary classification task, we assign a ground truth class label, WT-like or LoF, to each variant by thresholding its R-WT activity. If it is less than 0.6, the variant is considered to be LoF, otherwise it is considered WT-like. The class labels thus obtained are identical to those from the data providers (Donnelly et al. 2021).

In this manner, out of the 28 variants, 13 were classified as WT-like, while the remaining 15 were classified as LoF (Fig. 2). We validated the ground truth classifications and the R-WT activity against known pathogenic and benign variants in ClinVar (2024-01-27) and HGMD (2021-04). Out of 28, 6 variants (p.F354L, p.R297S, p.A241P, p.D194Y, p.P179R, p.G163R) were known to be pathogenic (P/LP or DM) or benign (B/LB) without any conflicting information. The ground truth classifications for these variants were consistent with the clinical assertions; i.e., all pathogenic variants were labeled as LoF and all benign variants as WT-like. The assays were therefore considered reliable.

### Evaluation set

Since 6 variants (p.F354L, p.R297S, p.A241P, p.D194Y, p.P179R, p.G163R) out of the 28 were known to be pathogenic or benign without conflicting information in clinical databases, we removed them from our final evaluation set, to ensure that the evaluation set does not include variants possibly used to train the predictors. There were 14 other variants in the clinical databases that were either annotated as a VUS in ClinVar, a DM? in HGMD or had conflicting information and consequently were retained in the evaluation set.

Since many tools are developed primarily for predicting the effects of missense variants, we also investigated performance on a reduced evaluation set, obtained by the removal of p.K84del.

### Evaluation metrics

To evaluate the predictors, we considered two sets of metrics for (1) R-WT activity prediction and (2) predicting the ground truth class label (WT-like or LoF). For the R-WT activity prediction, we used Pearson’s correlation and Kendall’s Tau, as standard performance metrics for regression. For the binary class label prediction, we used the area under the ROC curve (AUC).

Since the submission guidelines explicitly elicited predictions for R-WT activity, the predictions from the submitted model were used unaltered for computing Pearson’s correlation and Kendall’s Tau. However, since computing AUC requires a prediction score for which a higher (lower) value corresponds to the positive (negative) class, LoF (WT-like), we negated the predictions (multiplying by $-1$) for the AUC computation. The same approach was adopted for the Experimental-Max predictor; see “Experimental-Max”. Since all baseline predictors were built to give a higher value for function disruption or pathogenicity and were calibrated as a probability between 0 and 1, we transformed their output, $\hat{y}$, to $1 - \hat{y}$ for computing Pearson’s correlation and Kendall’s Tau. Their unaltered output was used to compute AUC.

If a tool did not predict on a variant, we replaced each missing prediction with an average of the prediction made on all other variants. This allowed evaluation of all tools on the same set of variants; i.e., the entire evaluation set, and consequently, ensured a fair comparison.

### Uncertainty quantification

We calculated each performance metric on 1000 bootstrap variant sets created from the evaluation set by sampling with replacement (Efron and Tibshirani 1986). Each bootstrap variant set was of the same size as the evaluation set, i.e., containing 22 or 21 variants for the analysis with and without p.K84del, respectively. Each bootstrap variant set for calculating the classification metrics was obtained with stratified sampling across WT-like and LoF variants and sampling with replacement within the two groups. This ensured that the number of LoF and WT-like variants was identical to that in the evaluation set. Each bootstrap variant set for calculating the regression metrics was obtained with standard sampling with replacement from the evaluation set.

**Fig. 3:**
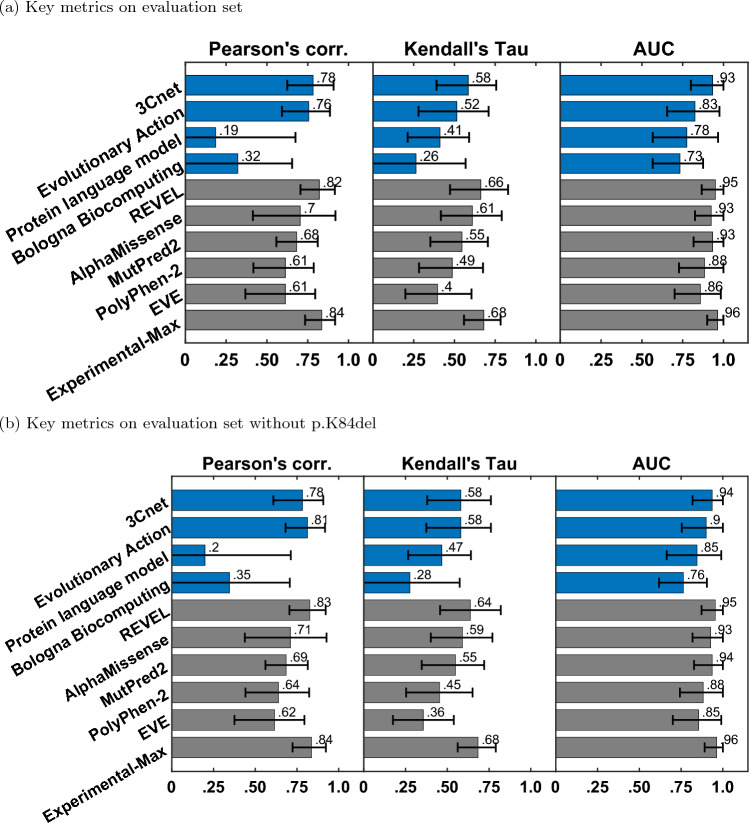
Pearson’s correlation, Kendall’s Tau, and area under the ROC curve (AUC) for submitted methods (blue), publicly available tools (grey) as baselines, and Experimental-Max (grey). The error bars correspond to the 5th and 95th percentiles computed with 1000 bootstrap samples. Only the best-performing method from each team is displayed. The submitted methods are shown in order of their average ranks on the three metrics. The baselines are also shown in the order of their average ranks and are ranked separately from the submitted methods

**Fig. 4:**
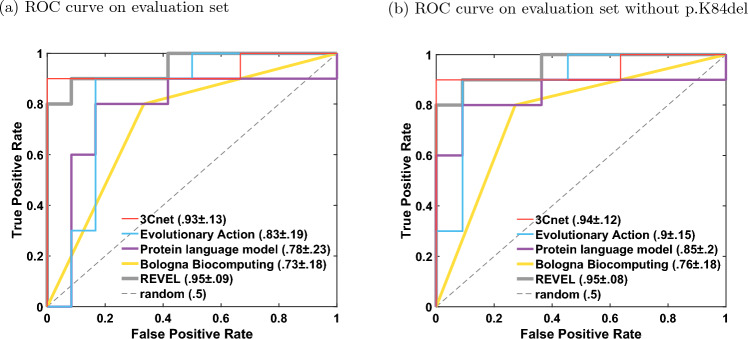
The receiver operating characteristic (ROC) curves for the best-performing model for each team and the best baseline model REVEL. AUC values are shown along with 1.96 $\times$ standard deviation from their bootstrap estimates

In this manner, we obtained 1000 bootstrap estimates of each metric. In Fig. 3 and Table 4 we show the 90% confidence interval for each metric, obtained from the 5th and 95th percentile of its bootstrap estimates. In Fig. 4 we provide a Gaussian approximation based 95% confidence interval for the AUC values as the $1.96\times$ standard deviation derived from its bootstrap estimates.

**Table 4:** Performance of the best model from each participating team, publicly available baseline models and Experimental-Max along with 90% confidence interval

Methods	Pearson’s correlation	Kendall’s Tau	AUC
	[5%, 95%]	[5%, 95%]	[5%, 95%]
3Cnet	0.783 [0.624, 0.909]	0.584 [0.390, 0.757]	0.933 [0.800, 1.000]
Evolutionary action	0.756 [0.592, 0.886]	0.515 [0.280, 0.710]	0.825 [0.654, 0.975]
Protein language model	0.186 [$-$ 0.093, 0.675]	0.411 [0.213, 0.590]	0.775 [0.567, 0.967]
Bologna biocomputing	0.321 [$-$ 0.064, 0.655]	0.264 [$-$ 0.056, 0.569]	0.733 [0.567, 0.875]
REVEL	0.821 [0.705, 0.916]	0.662 [0.473, 0.829]	0.950 [0.867, 1.000]
AlphaMissense	0.704 [0.414, 0.920]	0.610 [0.417, 0.790]	0.925 [0.825, 1.000]
MutPred2	0.682 [0.557, 0.811]	0.547 [0.352, 0.705]	0.933 [0.817, 1.000]
PolyPhen-2	0.613 [0.417, 0.786]	0.487 [0.283, 0.675]	0.883 [0.727, 1.000]
EVE	0.613 [0.368, 0.796]	0.396 [0.199, 0.605]	0.858 [0.700, 0.983]
Experimental-Max	0.836 [0.734, 0.917]	0.681 [0.558, 0.784]	0.964 [0.900, 1.000]

Participant models are listed in the order of their rankings. Baseline models are ranked separately, and also listed in order of their rankings

### Ranking

The methods were ranked based on their performance on three metrics: Pearson’s correlation, Kendall’s Tau, and AUC. The predictors were first ranked based on each of the three metrics separately. The final rank of a predictor was obtained by averaging its ranks over the three metrics. The ranking was performed first between the predictors submitted by each team separately to pick the best predictor from each team. The ranking was then performed between the representative predictors from all teams. The baseline predictors were ranked separately from the submitted predictors.

### Identification of difficult-to-predict variants

**Fig. 5:**
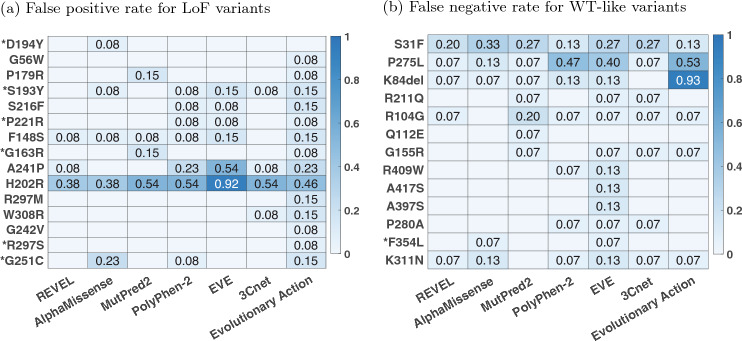
Difficult-to-predict variants and differences among competitive methods. All methods with an AUC above 0.8 were considered for this analysis. **a** The heatmap of the false positive rate of a method at each LoF variant; see “Difficult-to-predict variants”. **b** The heatmap of the false negative rate of a method at each WT-like variant. The variants with an asterisk are known pathogenic or benign variants in ClinVar and/or HGMD without any conflicting information

In Fig. 5, we quantify the difficulty in predicting each variant across predictors. For this analysis, we only incorporated the best predictor from each team (based on ranking) that also had an AUC above 0.8. Thus only 3Cnet and evolutionary action qualified based on this criteria. We additionally incorporated the publicly available baseline predictors (see “Models and baselines”) for this analysis, since all of them had an AUC greater than 0.8. For each predictor, we quantified the difficulty of predicting a LoF variant as the false positive rate (FPR) of the predictor when using the predicted value at the variant as a classification threshold. In other words, it is the fraction of variants in the WT-like set that were predicted to have a lower R-WT activity than the LoF variant at hand. Similarly, the difficulty in predicting a WT-like variant was quantified as the false negative rate (FNR) of the predictor based on the predicted value at the variant as the classification threshold; i.e., the fraction of variants in the LoF set that was predicted to have a higher R-WT activity than the WT-like variant at hand. An LoF (or WT-like) variant consistently having a high FPR (or FNR) across predictors is considered to be a difficult-to-predict variant. In this analysis, we considered all 28 variants, including those that were removed from the evaluation set for comparing predictors.

### Clinical variant classification

Only 4 out of the 28 variants considered in this work are clinically actionable with a definitive ClinVar classification of P/LP or B/LP. To investigate if the remaining 24 variants could be moved to more definitive categories, we collected and combined the evidence available for each variant under the American College of Medical Genetics and Genomics (ACMG) and the Association for Molecular Pathology (AMP) variant classification guidelines for rare genetic disease diagnosis (Richards et al. 2015). Precisely, we considered the functional assay results, computational evidence, allele frequency from population data and evidence from other co-located pathogenic variants by applying evidence codes PS3/BS3, PP3/BP4, PM2/BS1 and PM5, respectively. The original guidelines interpreted each evidence type on an ordinal scale of *supporting, moderate, strong* and *very strong* and provided rules to combine evidence strength to make pathogenic (P or LP) or benign (B or LB) assertion. For example, 1 strong, 2 moderate, and 2 supporting lines of evidence lead to P, whereas 2 moderate and 2 supporting lines lead to LP. The recently developed point-based system for variant interpretation (Tavtigian et al. 2020) assigned points to each strength level: supporting, moderate, strong, and very strong evidence towards pathogenicity (benignity) correspond to 1 ($-1$), 2 ($-2$), 4 ($-4$) and 8 ($-8$) points, respectively. Here we use the point scale, under which a P, LP, VUS, LB, or B assertion is made if the total points from the evidence collected for a variant were in the range $\ge 10$, [6, 9], [0, 5], $[-6, -1]$ or $\le -7$, respectively. The VUS category is further divided into VUS-low, VUS-mid and VUS-high categories corresponding to the range [0, 1], [2, 3] and [4, 5], respectively.

Following the point-based system, if a variant was determined to be LoF from an assay’s output, we applied the PS3 code with 1 point (supporting for pathogenicity), whereas if it was determined to be WT-like, we applied the BS3 code with $-1$ point (supporting for benignity). Combining the results from the luciferase and the gel-shift assay, this approach resulted in giving 2 points for each variant annotated as LoF by both assays, and $-2$ points for each variant annotated as WT-like by both assays. In the case of p.H202R where the two assays disagree, 1 point from the luciferase assay and $-1$ point from the gel-shift assay led to a net score of 0 points. We incorporated population data evidence by looking at a variant’s allele frequency from healthy controls in gnomAD v4.1 where there are 5 P/LP variants, each with allele count of 1. All variants considered in this work were either absent from gnomAD v4.1 or were found with very low allele frequency (AF $\lessapprox 10^{-5}$), except for p.F354L with AF = 0.0051. Peutz–Jeghers syndrome (PJS) being an autosomal dominant trait, we applied PM2 only for variants absent from gnomAD as per the guidelines (Richards et al. 2015). Instead of applying PM2 as a moderate level evidence, as recommended by the original guidelines, we applied PM2 at a supporting level with 1 point based on the recent updates to the guidelines (ClinGen SVI Working Group 2019). For BS1 we used an allele frequency of 0.001 as a threshold above which the the code was applied. Thus only p.F354L qualified for BS1 with $-4$ points (strong benignity). The remaining variants present in gnomAD v4.1 with an allele frequency less than 0.001 were considered to have indeterminate evidence. Consequently, no evidence code was applied for these variants.

To quantify the computational evidence on the point scale, we used REVEL scores and applied the recently derived score intervals, corresponding to the evidence strength (Pejaver et al. 2022). Precisely, if the score for a variant was in the interval [0.644, 0.773), [0.773, 0.932) and [0.932, 1], PP3 was applied as supporting, moderate and strong, with 1, 2 and 4 points, respectively, whereas if the score was in the interval (0.183, 0.290], (0.016, 0.183] and (0.003, 0.016], BP4 was applied as supporting, moderate, strong, with $-1$, $-2$ and $-4$ points, respectively. Other P and LP ClinVar variants at the same amino acid position were considered as evidence (PM5) if the REVEL score rounded to 2 decimal places of the tested variant was equal to or higher than the REVEL score of the co-located P and LP variants, with 2 points for the first P variant, 1 point for the first LP variant, and 1 point for any additional P or LP variant. Other B and LB variants at the same position were also considered but none were identified. Since the variants were obtained from cancer biopsies and not PJS cases, no case data was available for this study and consequently, de novo counts (PM6/PS2) or segregation data (PP1) was not considered.

## Models and baselines

The participant teams used a diverse set of approaches in terms of the features, machine learning models and training datasets; see Table 1. The top performing model, 3CNet, an improved version of the base model from Won et al. (2021), used structure, conservation, and physical and biochemical features. A long short-term memory (LSTM) (Hochreiter and Schmidhuber 1997) network, trained on simulated variants from conservation data from UniRef (Suzek et al. 2007), was used as a feature extractor. A random forest model based on the extracted features was then trained on variants from ClinVar (Landrum et al. 2016) and gnomAD (Karczewski et al. 2020); see Supplementary File 2. The second best-performing method, evolutionary action, is based on a mathematical model for the action of coding mutations on fitness. Protein language model was based on Bidirectional encoder representations from transformers (Devlin et al. 2019) (BERT) trained on Pfam (Mistry et al. 2021) representative proteome domain sequence data. Bologna Biocomputing created a meta predictor from three $\Delta \Delta$G predictors, INPS3D (Savojardo et al. 2016), PoPMuSiC 2.1 (Dehouck et al. 2011) and FOLDEF (Guerois et al. 2002), and a sequence based residue solvent exposure predictor, DeepREx (Manfredi et al. 2021).

In addition to evaluating the submitted predictors, we also evaluated publicly available tools PolyPhen-2 (Adzhubei et al. 2010), REVEL (Ioannidis et al. 2016), MutPred2 (Pejaver et al. 2020), EVE (Frazer et al. 2021), and AlphaMissense (Cheng et al. 2023). These tools were selected to represent the diversity of variant effect predictors, each employing distinct machine learning techniques. PolyPhen-2, one of the earliest variant effect predictors, uses a simpler Naive Bayes model. MutPred2 and REVEL have consistently ranked among the top performing methods across multiple coding variant challenges in CAGI (The Critical Assessment of Genome Interpretation Consortium 2024). MutPred2 is an ensemble of shallow, fully connected neural networks trained from scratch while REVEL is a meta predictor that combines 18 prediction scores from 13 computational tools. In contrast to PolyPhen-2, MutPred2 and REVEL, which all rely on traditional supervised learning approaches, EVE and AlphaMissense rely on more modern techniques: EVE employs a generative variational autoencoder, and AlphaMissense uses a language model. These tools are trained in an unsupervised and/or weakly supervised manner, without reliance on pathogenicity or functional effect labels.

### Experimental-Max

We derive an Experimental-Max predictor that incorporates the assay replicates to quantify its consistency and also the maximum achievable performance on all three metrics. The biological and technical replicates capture the variability of the assay in measuring the R-WT activity. We use the average R-WT activity across the replicates as the ground truth for evaluation; see “Ground truth for evaluation”. High variability of the replicates around the average indicates low consistency of the assay. Experimental-Max’s predicted R-WT activity on a variant is determined by first randomly picking a biological replicate in which it appears, and then using the R-WT activity of a randomly picked technical replicate within the biological replicate. Unlike conventional predictors, Experimental-Max is stochastic; i.e., has randomness in its output. Thus repeating the sampling is likely to give a different predicted R-WT activity for the variant. Consequently, the performance measured with Experimental-Max predictions over a set of variants is also stochastic. To obtain a robust estimate of a performance metric, we generated 1000 Experimental-Max predictors by resampling and averaged the performance computed over them. The confidence interval for Experimental-Max’s performance in Fig. 3 and Table 4 is obtained as the 5th and 95th percentile of the 1000 estimates. Pearson corr. and Kendall’s Tau computed for Experimental-Max quantifies the consistency of the assay in measuring R-WT activity, whereas its AUC quantifies the consistency in separating LoFs from WT-like variants. Experimental-Max performance additionally serves as an upper limit to a predictor’s performance, since a predictor can not be expected to predict the assay output better than the replicates. The small gap between a predictor’s performance and Experimental-Max suggests that a predictor is comparable to the assay in estimating the true R-WT activity of the variants and separating LoFs from WT-like variants.

## Results

### Performance of submitted predictors

We evaluated the participant team models based on their performance on Pearson’s correlation, Kendall’s Tau, and AUC, computed on the evaluation set of 22 variants. The best-performing predictor from each team was first selected based on the three metrics as the top-ranking predictor from the team; see “Ranking”. The best-performing predictors from each team were then re-ranked based on the three metrics; see Figs. 3a and 4a, and Table 4.Among the four participant team models, 3Cnet performed the best on all three metrics: Pearson’s corr = 0.78, Kendall’s Tau = 0.58, and AUC = 0.93. Evolutionary action performed the second best: Pearson’s corr. = 0.76, Kendall’s Tau = 0.52 and AUC = 0.83. The performance of 3Cnet was better than evolutionary action with statistical

significance on all three metrics. Statistical significance was determined using a one-sided binomial test with a number of wins on 1000 bootstrap samples as the test statistic. 3Cnet won 629, 687, and 838 times on Pearson’s corr., Kendall’s Tau and AUC, respectively, giving p-values less than $10^{-16}$, $10^{-32}$, and $10^{-110}$, respectively. The p-value was computed as the probability that the Binomial(0.5, 1000) variable is greater than or equal to the number of wins.

All participant models demonstrated improved performance to varying degrees on the reduced evaluation set (removing p.K84del) containing only missense variants; see Figs. 3a and 4b. Performance of evolutionary action, Protein language model, and Bologna biocomputing improved significantly on all three metrics, whereas 3Cnet only improved on AUC by a small margin. In fact, evolutionary action performed better than 3Cnet on Pearson’s correlation (0.81 vs. 0.78) and identically on Kendall’s Tau (0.581 vs. 0.581). 3Cnet retained its advantage on AUC at 0.94 vs. 0.9 for evolutionary action. The significant improvement in evolutionary action’s performance on the removal of p.K84del was observed because it predicted the indel as having the lowest R-WT activity in the evaluation set, whereas it retains enough activity to be deemed WT-like as per both assays. The ROC curves of evolutionary action and Protein language model depicted improved behavior upon the removal of p.K84del since they no longer demonstrate a false positive error at 0 true positive rate; see Fig. 4.

### Comparison with publicly available tools

We also evaluated the performance of publicly available tools, REVEL, AlphaMissense, MutPred2, PolyPhen-2, and EVE on the evaluation set as a baseline; see Figs. 3a and 4a, and Table 4. REVEL was the top performing tool on all three performance metrics when compared to other publicly available tools and the submitted predictors: Pearson’s correlation = 0.82, Kendall’s Tau = 0.66 and AUC = 0.95. Its improvement over the best-performing participant model 3Cnet was significant on all three metrics with 720 (Pearson’s correlation), 801 (Kendall’s Tau) and 527 (AUC) wins, and p-values $<10^{-45}$, $<10^{-86}$ and $=0.041$, respectively. AlphaMissense was the second-best-performing tool. The top performing submitted predictor, 3Cnet, performed better than AlphaMissesnse on Pearson’s correlation and AUC, but not on Kendall’s Tau; see Fig. 3a and Table 4.

The performance of most publicly available tools appeared more or less similar with and without p.K84del (except correlations measured for PolyPhen-2 and EVE); see Figs. 3b and 4b. However, this could be an artifact of our imputation approach. Most publicly available tools (except PolyPhen-2^1^) did not make predictions on p.K84del. For a fair comparison with participant models on the same set of variants, a prediction score for p.K84del was imputed using the average over other variants without missing predictions. Since p.K84del’s R-WT activity was in the intermediate range, the average-based imputation approach worked in favor of the tools and p.K84del’s inclusion in the evaluation set did not affect their performance adversely, unlike evolutionary action, Protein language model, and Bologna biocomputing. The lower performance of EVE could be attributed to missing predictions for three other variants (p.A397S, p.R409W, p.A417S), in addition to p.K84del, which were also imputed by the average prediction.

### Comparison with Experimental-Max

The consistency of the assay was quantified by evaluating the Experimental-Max predictor. At Pearson’s corr. = 0.83 and Kendall’s Tau = 0.68, the assay demonstrated medium level of consistency in measuring the R-WT activity; see Fig. 3a. The consistency was high in separating LoFs from WT-like variants at AUC = 0.96. In addition to quantifying assay consistency, Experimental-Max performance on the three metrics gave upper limits to a predictor’s performance, since a predictor can not be expected to better predict the assay output than the assay replicates. REVEL comes very close to Experimental-Max in its performance; with a gap of ${\sim }0.01$ on AUC, and ${\sim }0.02$ on Pearson’s correlation and Kendall’s Tau. The trend holds true even after the removal of the imputed variant p.K84del with a slightly worse gap of ${\sim }0.04$ on Kendall’s Tau. The gap between the performances of 3Cnet, AlphaMissense, and MutPred2 with Experimental-Max is not too large either in terms of AUC (${\sim }0.03$). Overall, the comparison between the top performing models and Experimental-Max reveals that these predictors are comparable to the assay in terms of correlation with STK11 variants’ R-WT activity and separating LoFs from WT-like variants. However, an evaluation on a larger set of variants might be necessary to confidently assert this claim.

### Difficult-to-predict variants

In Fig. 5, we quantify the difficulty in predicting each variant over a set of competitive predictors having an AUC greater than 0.8. For a LoF (or WT-like) variant the difficulty is quantified as the FPR (or FNR) of each selected predictor at that variant; see “Identification of difficult-to-predict variants”. Some LoF variants (e.g., p.R297S, p.G242V, p.G56W, p.D194Y) are easy to predict by most methods. All LoFs, except p.H202R, had at least one method predicting lower activity than all WT-like variants, i.e., FPR = 0. Variant p.H202R was significantly difficult to predict as LoF by all predictors. Some WT-like variants (e.g., p.Q112E, p.A417, p.A397S) were easy to predict for most predictors. All WT-like variants, except p.S31F and p.P275L, had at least one method predicting higher activity than all LoF variants, i.e., FNR = 0. The difficulty in predicting p.S31F and p.P275L can be explained by the observation that they have the lowest experimental R-WT activity values among all WT-like variants. Furthermore, in case of p.P275L multiple predictors have FNR as low as 0.07.

The LoF variant p.H202R has an average FPR of 0.537 across the selected predictors, indicating that over half of the benign variants were predicted to have a lower R-WT activity on average. Thus it is an outlier LoF variant that is not predicted well by any competitive predictors. Donnelly et al. (2021) also made a similar observation based on the predictive tools considered in their assessment. Compared to other LoF variants, p.H202R has a higher R-WT activity of 0.32; only five (p.R297M, p.W308R, p.G242V, p.R297S, p.G251C) out of the fifteen LoF variants have a higher R-WT activity. However, despite their higher activity, the five variants are well predicted by multiple predictors, suggesting that the activity level of p.H202R does not explain the challenging nature of the variant. Interestingly, p.H202R is the only variant where the two assays differ in their classification. It is annotated LoF based on the luciferase assay and WT-like based on the gel-shift assay (Donnelly et al. 2021); see Table 3. p.H202R is located in functional regions VIB-VIII (amino acids 172–225), a part of the kinase domain specifically related to substrate recognition (Hearle et al. 2006), which affects its binding affinity to p53, but not its kinase activity. Since the luciferase assay measures an STK11 variant’s effect on the transcriptional activity of p53, it shows a reduced activity due to p.H202R. It is likely that the predictors perform well concerning the kinase activity prediction, but fail to capture p.H202R’s effect on binding p53.

**Fig. 6:**
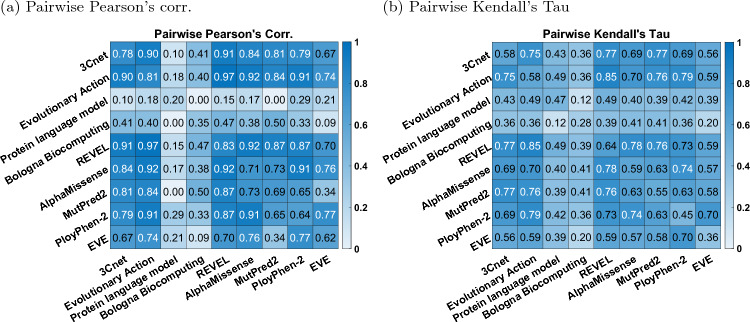
Correlation between predictors. **a** Pearson’s corr., **b** Kendall’s Tau. Each off-diagonal element gives the pairwise correlation between a pair of predictors. The diagonal elements give correlation between the predictor and the experimental R-WT activity for comparison. p.K84del was excluded while computing the correlations in this figure, since most publicly available tools do not make prediction on indels

### Correlation between predictors

Computing the pairwise correlation between the predictors, we observed that the top-performing predictors were more correlated with each other, compared to the correlation with the experimentally measured R-WT activity; see Fig. 6. This trend has been observed previously in many CAGI challenges (The Critical Assessment of Genome Interpretation Consortium 2024; Clark et al. 2019) and can be attributed to the predictors using similar features and training data.

### Clinical variant classification

Performing variant classification by combining the newly available functional evidence from the assays with computational evidence from REVEL, population data evidence from gnomAD and evidence from co-located pathogenic variants (see “Clinical variant classification”) revealed that 16 new variants could attain a clinically actionable LP (6 variants) or LB (10 variants) classification for the diagnosis of Peutz–Jeghers syndrome (PJS); see Table 5. The variants that received a definitive classification included 1) 15 variants from the evaluation set whose clinical significance was unknown since they were either not observed in ClinVar and HGMD or were observed as DM? in HGMD or were observed in ClinVar as VUS or with conflicting annotations, 2) 1 variant observed in HGMD as disease-causing mutation (DM), but were absent from ClinVar and 3) 4 variants already observed in ClinVar with a definitive classification of either P/LP or B/LB. Although there were 8 variants that failed to receive a definitive classification, 5 (3) of them attained a VUS-high (VUS-low) status, moving them closer

to LP (LB) classification, thereby reducing the uncertainty in their pathogenicity/benignity status. The 4 variants, already having definitive classification in ClinVar, received consistent classifications based on the four evidence types. However, 3 out of the 4 variants were deposited with stronger total evidence in ClinVar as they had P (p.R297S) or P/LP (p.D194Y) classification instead LP or B/LB (p.F354L) classification instead of LB. Such differences in the classification are expected since we do not consider all types of evidence (e.g., case data) allowed by the clinical guidelines. Thus the total points and the clinical classifications (Final category) for other variants given in Table 5 might change slightly if other types of evidence are also considered.

**Table 5:** Clinical variant classification based on population data, functional assay results, computational predictions and other co-located pathogenic variants; see “Clinical variant classification” for details

Variant	ClinVar	HGMD	Population data evidence		Functional assay evidence		Computational evidence		Evidence from co-located pathogenic variants		Total points	Final category
			gnomAD AF	Code (points)	Assay result	Code (points)	REVEL score	Code (points)	Variant-P/LP (REVEL score)	Code (points)		
G56W				PM2 (1)	LoF-LoF	PS3 (2)	0.932	PP3 (4)			7	LP
P179R		DM		PM2 (1)	LoF-LoF	PS3 (2)	0.939	PP3 (4)	P179Q-LP (0.94)	PM5 (1)	8	LP
S193Y				PM2 (1)	LoF-LoF	PS3 (2)	0.871	PP3 (2)			5	VUS- high
S216F	VUS			PM2 (1)	LoF-LoF	PS3 (2)	0.954	PP3 (4)			7	LP
P221R				PM2 (1)	LoF-LoF	PS3 (2)	0.892	PP3 (2)			5	VUS-high
F148S				PM2 (1)	LoF-LoF	PS3 (2)	0.728	PP3 (1)			4	VUS-high
A241P		DM		PM2 (1)	LoF-LoF	PS3 (2)	0.782	PP3 (2)			5	VUS-high
R297M				PM2 (1)	LoF-LoF	PS3 (2)	0.936	PP3 (4)	R297S-P (0.94)	PM5 (2)	9	LP
H202R	VUS		3.16E−06		LoF-WT		0.424				0	VUS-low
W308R	VUS		.	PM2 (1)	LoF-LoF	PS3 (2)	0.881	PP3 (2)	W308C-LP (0.73)	PM5 (2)	7	LP
									W308L-LP (0.84)			
G242V		DM?		PM2 (1)	LoF-LoF	PS3 (2)	0.97	PP3 (4)	G242R-P(0.97)	PM5 (2)	9	LP
G251C				PM2 (1)	LoF-LoF	PS3 (2)	0.833	PP3 (2)			5	VUS-high
S31F				PM2 (1)	WT-WT	BS3 ($-2$)	0.8	PP3 (2)			1	VUS-low
P275L	VUS			PM2 (1)	WT-WT	BS3 ($-2$)	0.663	PP3 (1)			0	VUS-low
K84del	VUS		4.34E−06		WT-WT	BS3 ($-2$)					$-2$	LB
R211Q	VUS/LB		1.81E−05		WT-WT	BS3 ($-2$)	0.215	BP4 ($-1$)			$-3$	LB
Q112E	VUS		1.24E−06		WT-WT	BS3 ($-2$)	0.393				$-2$	LB
G155R	VUS		1.90E−06		WT-WT	BS3 ($-2$)	0.382				$-2$	LB
R104G	VUS		3.72E−06		WT-WT	BS3 ($-2$)	0.582				$-2$	LB
R409W	VUS/LB	DM?	4.28E−05		WT-WT	BS3 ($-2$)	0.238	BP4 ($-1$)			$-3$	LB
A417S	VUS		1.02E-05		WT-WT	BS3 ($-2$)	0.102	BP4 ($-2$)			$-4$	LB
A397S	VUS/LB	DM?	1.87E−05		WT-WT	BS3 ($-2$)	0.03	BP4 ($-2$)			$-4$	LB
P280A	VUS			PM2 (1)	WT-WT	BS3 ($-2$)	0.097	BP4 ($-2$)			$-3$	LB
K311N				PM2 (1)	WT-WT	BS3 ($-2$)	0.461				$-1$	LB
D194Y*	P/LP	DM	.	PM2 (1)	LoF-LoF	PS3 (2)	0.929	PP3 (2)	D194H-P (0.94)	PM5 (4)	9	LP
									D194V-P (0.94)			
									D194E-P (0.84)			
G163R*	LP	DM		PM2 (1)	LoF-LoF	PS3 (2)	0.933	PP3 (4)	G163D-P (0.95)		7	LP
R297S*	P			PM2 (1)	LoF-LoF	PS3 (2)	0.936	PP3 (4)			7	LP
F354L*	B/LB	DM?	5.10E−03	BS1 (-4)	WT-WT	BS3 ($-2$)	0.156	BP4 ($-2$)			$-8$	B

Variants with an asterisk were already classified as pathogenic or benign in ClinVar (2024-01-27) (Landrum et al. 2016)

## Discussion

The performance levels of the top methods in the STK11 challenge were on the higher end compared to the previous biochemical effect challenges in CAGI (The Critical Assessment of Genome Interpretation Consortium 2024). However, since the evaluation was performed on a small set of 22 variants, it is possible that the performance may not generalize to the same extent on other STK11 variants. Evaluation of a larger set of variants would be necessary to confidently characterize the performance of computational predictors on kinase activity prediction. Assuming that the results would indeed generalize, the high level of performance on STK11 variants is partly because of advancements in machine learning and partly because the enzymatic activity of STK11 might be easier to predict computationally compared to other biochemical effects/genes. The latter can be justified by the observation that the improvement in the performance for STK11 is also observed for well-characterized tools such as MutPred2, evolutionary action, and PolyPhen-2; see NAGLU and PTEN challenge results in The Critical Assessment of Genome Interpretation Consortium (2024).

The top-ranking submitted method, 3Cnet, performed competitively with REVEL, the best-performing method overall. Interestingly, 3Cnet, based on modern deep learning approaches and LSTM architecture, with innovative use of simulated variants, is a simpler predictor compared to REVEL, a meta predictor that combines 13 other predictors in an ensemble.

The predictors were consistent on some LoF variants while differed on other LoF variants. All predictors fail to predict the effect of p.H202R (LoF) on p53’s transcriptional activity, as measured by the luciferase assay. There is evidence suggesting that p.H202R might only affect STK11’s ability to bind with p53 and not its ability to function as a kinase; see “Difficult-to-predict variants”. It is likely that the tools are well correlated with STK11’s kinase activity overall but fail to capture the p.H202R’s role in binding p53. Our variant classification analysis remained inconclusive towards establishing the pathogenicity/benignity of p.H202R.

A unique feature of the STK11 challenge was the presence of multiple biological and technical replicates in the data generation process, compared to similar CAGI challenges where only technical replicates were available (The Critical Assessment of Genome Interpretation Consortium 2024). We incorporated the replicates in an Experimental-Max predictor to quantify assay consistency and derive an upper bound to the predictive performance. The assay demonstrated medium consistency on the correlation metrics and high consistency on AUC. Multiple models reached AUC levels close to the maximum achievable AUC from Experimental-Max. REVEL also reached close to the maximum performance on Pearson’s corr. and Kendall’s Tau. The STK11 challenge is the first instance in CAGI to demonstrate that the computational tools could separate LoFs from WT-like variants and predict enzyme activity at a precision comparable to the assay, although a larger set of variants and more replicates are necessary to investigate this hypothesis thoroughly.

Our variant classification analysis justified clinical actionability on 16 variants (6 LP and 10 LB) that were previously had uncertain significance. This further highlights the importance functional studies and computational tools for improved variant classification when other types of evidence such as segregation data and prevalence in patients are not available or give inconclusive results (Table 5).

## Supplementary Information

Below is the link to the electronic supplementary material.

Supplementary file 1File S1. AUC of the participant models and baseline predictors on the gel shift assay (pdf 59 KB)

Supplementary file 2File S2. A zip file containing spreadsheets for 1) raw assay output with replicates, 2) all participant teams’ model predictions, 3) baseline models from publicly available tools. Additional documents provided by the participant teams, describing their models, are also included (zip 219 KB)

## Data Availability

The raw assay output with replicates, participant and baseline model predictions are available in supplementary File 8.

## Footnotes

1 A prediction for p.K84A was used by the tool as substitute for p.K84del.

## Code availability

Not relevant.
