# Supplementary material for: Evaluating predictors of kinase activity of STK11 variants identified in primary human non-small cell lung cancers
Source: Hum Genet. 2025 Feb 12;144(2-3):127–42. doi: 10.1007/s00439-025-02726-0 (PMC11976797; doi:10.1007/s00439-025-02726-0)
Supplement: Supplementary file 1 — File S1. AUC of the participant models and baseline predictors on the gel shift assay (pdf 59 KB) [file 439_2025_2726_MOESM1_ESM.pdf]

Performance evaluation for the gel shift assay.

(a) Area under the ROC curve (AUC) on Evaluation set

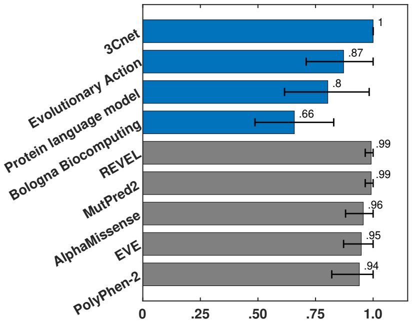

(b) The receiver operating characteristic (ROC) curves

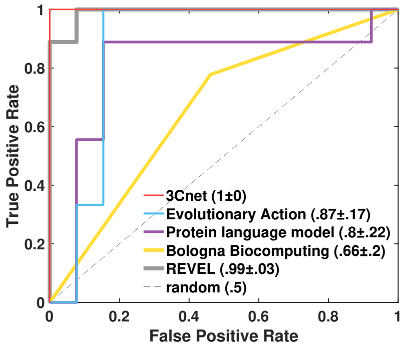

Figure 1: Evaluation results based on the autophosphorylation assay result.

Table 1: Predictor performance evaluation results.

Measures	AUC [5%, 95%]
3Cnet	1.000 [1.000, 1.000]
Evolutionary Action	0.872 [0.709, 1.000]
Protein language model	0.803 [0.615, 0.983]
Bologna Biocomputing	0.658 [0.487, 0.829]
REVEL	0.991 [0.966, 1.000]
MutPred2	0.991 [0.966, 1.000]
AlphaMissense	0.957 [0.880, 1.000]
EVE	0.949 [0.872, 1.000]
PolyPhen-2	0.940 [0.821, 1.000]
