# Supplementary material for: Evaluating predictors of kinase activity of STK11 variants identified in primary human non-small cell lung cancers
Source: Hum Genet. 2025 Feb 12;144(2-3):127–42. doi: 10.1007/s00439-025-02726-0 (PMC11976797; doi:10.1007/s00439-025-02726-0)
Supplement: Supplementary file 2 — File S2. A zip file containing spreadsheets for 1) raw assay output with replicates, 2) all participant teams’ model predictions, 3) baseline models from publicly available tools. Additional documents provided by the participant teams, describing their models, are also included (zip 219 KB) [file 439_2025_2726_MOESM2_ESM.zip › Supplementary File S2/Bologna Biocomputing description.pdf]

MATERIAL AND METHODS

Bologna Biocomputing Group

STK11 challenge, Submission 1

We use as a reference of STK11 the structure of the heterotrimeric LKB1-STRADalpha-MO25alpha complex (PDB: 2WTK, Zeqiraj et al. 2009) where we analyse the active site, the heterotrimeric interface, the phosphorylation sites. Our functional annotation is based on the conservation of the chemical-physical properties of residues when critical for protein functionality, their steric hindrance, and the property of residues to be solvent accessible or not. Furthermore, we based our annotations on the effect of variations on protein stability as obtained adopting a consensus of three state-of-the-art ΔΔG predictors: INPS3D (Savojardo et al. 2016), PoPMuSiC 2.1 (Dehouck et al. 2011), and FoldX (Guerois et al. 2002).

We used DeepREx-WS (Manfredi et al. 2021, https://deeprex.biocomp.unibo.it, submitted), a deep-learning based method, for the prediction of residue solvent exposure starting from protein sequence.

References

Dehouck Y, Kwasigroch JM, Gilis D, Rooman M. (2011) *PoPMuSiC 2.1: a web server for the estimation of protein stability changes upon mutation and sequence optimality.* BMC Bioinformatics. 12:151

Guerois R, Nielsen JE, Serrano L. (2002) *Predicting Changes in the Stability of Proteins and Protein Complexes: A Study of More Than 1000 Mutations*. Journal of Molecular Biology, 330(2): 369-387

Savojardo C, Fariselli P, Martelli PL, Casadio R. (2016) *INPS-MD: a web server to predict stability of protein variants from sequence and structure.* Bioinformatics. 32(16):2542-4.

Zeqiraj E, Filippi BM, Deak M, Alessi DR, van Aalten DM. (2009) *Structure of the LKB1-STRAD-MO25 complex reveals an allosteric mechanism of kinase activation.* Science. 2009 Dec 18;326(5960):1707-11.
