# Supplementary material for: Evaluating predictors of kinase activity of STK11 variants identified in primary human non-small cell lung cancers
Source: Hum Genet. 2025 Feb 12;144(2-3):127–42. doi: 10.1007/s00439-025-02726-0 (PMC11976797; doi:10.1007/s00439-025-02726-0)
Supplement: Supplementary file 2 — File S2. A zip file containing spreadsheets for 1) raw assay output with replicates, 2) all participant teams’ model predictions, 3) baseline models from publicly available tools. Additional documents provided by the participant teams, describing their models, are also included (zip 219 KB) [file 439_2025_2726_MOESM2_ESM.zip › Supplementary File S2/3Cnet description.docx]

**Methods**

Our methods are based on the method of our previous research (Won et al., 2021). The baseline model of 3Cnet (Won et al., 2021) was improved by the following modifications. First, the secondary structures and solvent accessibility of the protein sequences predicted by DSSP (Joosten et al., 2010) and PSIPRED (McGuffin et al., 2000) were used as additional features when training the feature extractor. Also, unlike the original 3Cnet which only used neural networks to predict pathogenicity, transfer learning was applied to extract features from neural networks, and then random forests model was built using those features. Neural networks were trained by the conservation data, while random forests were trained using variant data from ClinVar and gnomAD.

**1. Generation of clinical data from the ClinVar database**

  We first curated 73,822 missense mutations in ClinVar, released in June 2021. For curation, we collected mutations of which molecular consequence was ‘missense variant’ and excluded mutations having not only zero review star but also unreliable review status such as ‘no assertion for the individual variant’, ‘no assertion criteria provided’, and ‘no assertion provided’. As our prediction algorithm makes use of protein sequences around mutation sites as input features, each variant was represented as the Human Genome Variation Society (HGVS) term, in which the transcript ID and the mutation information were given (den Dunnen et al., 2016). The canonical transcript ID in Refseq database, version GRCh37 (Pruitt et al., 2005), was referenced as transcript ID. Then, each missense variant was transformed into data representing a protein sequence composed of 201 amino acids centered around the variant position. Sequence data for both wild-type protein and mutant protein were generated to compare the difference in the context of amino-acid sequences.

  There are 5 labels for pathogenicity which are pathogenic (P), likely pathogenic (LP), variants with uncertain significance (VUS), likely benign (LB), and benign (B). To determine the pathogenicity of a single variant having conflict within multiple pathogenicity reports from ClinVar (e.g., pathogenic in one report while benign in the other report), those reports for the same variant were integrated and classified with following standard. When there are any reports saying a variant is pathogenic or likely pathogenic, we consider the variant is pathogenic except for the cases in which there are contrary reports. Similarly, a variant with any reports saying the variant is benign or likely benign is considered as a benign variant. We removed variants having contrary reports and variants of which reports are VUS only. As a result, we got 25,984 pathogenic variants and 47,838 benign variants from the ClinVar database. The input features of the clinical data are sequence representations of wild-type protein and mutant protein, while the output feature is a binary label for pathogenicity. Additionally, a subset of variants which were derived from MANE Select (https://www.ncbi.nlm.nih.gov/refseq/MANE/) transcripts was referred to canonical transcripts.

**2. Augmentation of clinical data with common variants from the gnomAD database**

  From the gnomAD database (Karczewski et al., 2020), we curated missense variants frequently observed in the general population, namely common variants. Found in the genome of a huge general population, such variants are thought to be benign and can be used to train predictors to get better precision by reducing false positives (Gilissen et al., 2012). We collected variants having allele frequency (AF) higher than 1% or 0.1 % and represented them as the HGVS term based on the canonical transcripts as we mentioned above. Only variants not included in the ClinVar database were curated as common variants to avoid conflicts and overfitting due to duplicate samples. In total, 27,376 variants, additionally 44,651 variants for AF 0.1% cutoff, were found to be common variants and their pathogenicity were all labeled as benign. The sequence data were also transformed into 201 AA representations around the mutation site.

**3. Generation of conservation data using multiple sequence alignment (MSA)**

  For 53,998 transcripts included in the RefSeq database, canonical protein MSA data were constructed to show the evolutionary conservation patterns of those proteins. For each transcript, the sequence was transformed into FASTA format and HHblits (Remmert *et al.*, 2012), a hidden markov model based algorithm, built MSA from the query sequence using UniRef30, version 2020.02, as a sequence database (Suzek *et al.*, 2007). These MSA results were utilized for two purposes: to derive input features of the networks and to generate simulated variants reflecting evolutionary constraints. Among the sequences aligned with the query sequence, only sequences having over 30% identity and 80% overlap with the query remained.

  145,849 variants collected from clinical data, including common variants as benign samples, were not sufficient to train a deep neural network, having more than 10,000 features as sequence inputs. Therefore, we generated simulated variants concerning amino acid frequency calculated at each residue in MSA data. If a residue is aligned with homologous sequences with over 50% frequency for a single amino acid, the residue is regarded as well conserved. Among simulated variants considering trinucleotide context, we defined the variants that had never been found at the well-conserved residue as pathogenic-like variants. On the other hand, the variants frequently found with the ratio higher than 10 % out of aligned amino acids and synonymous variants were defined as benign-like variants. We named these variants as general conservation data and utilized them to train the network. 1,838,905 pathogenic-like variants and 2,264,825 benign-like variants are transformed into sequence data just as ClinVar variants and common variants.

  Protein MSA data for NP_000446.1, the STK11 RefSeq canonical protein sequence, was used to extract STK11-specific pathogenic- and benign-like variants with the similar method we used to build general conservation data. To get the pathogenic-like variants with more strict standards compared to general conservation data, some variants were not regarded as pathogenic-like when amino acids with the similar physico-chemical properties are found in MSA at the residue. Amino acid properties can be positive-charged (Arg, His and Lys), negative-charged (Asp and Glu), polar-uncharged (Ser, Thr, Asn and Gln) and hydrophobic (Ala, Val, Ile, Lue, Met, Phe, Tyr and Trp). We also consider the size of the side chain to determine rigorous pathogenic-like variants of STK11. Those STK11 conservation variants (220 pathogenic, 686 benign) were also transformed into sequence data and utilized to train models.

**4. Network architecture of the pathogenicity predictor**

  The model network we built can be divided into two modules: a feature extractor and a pathogenicity classifier. The feature extractor is composed of two parallel layers utilizing long short-term memory (LSTM) networks, a type of RNN (Hochreiter and Schmidhuber, 1997). The first layer consists of bidirectional LSTM networks which independently featurize multiple input feature matrices: the wild-type sequence feature matrix, MSA feature matrix, mutant sequence feature matrix and structure feature matrix including secondary structure and solvent accessibility. The output feature matrices can conceive the context of the sequences through the recurrent networks to consider the influence of one amino-acid to other amino-acids. Then, the output matrix from wild-type sequence and that of MSA and structure feature matrices are merged to produce a concatenated feature matrix. Features for the same residue are concatenated so that the network can compare the amino-acid of the sequence with evolutionarily conserved amino-acids at that residue. output matrices of mutant sequence are also merged with MSA and structure feature matrices. Then, those two concatenated features (wild-type and mutant) are featurized once more using LSTM networks, remaining only the last feature vector for each recurrent network at this point. Finally, the output feature vector of the wild-type and the mutant sequences are concatenated to become an extracted feature vector.

  The pathogenicity classifier is composed of two fully connected (FC) layers. The first FC layer is expected to extract the difference between the wild-type and mutant sequences as a feature vector. Then, features from the SNVBox, a database providing features that predict the biological impact of single nucleotide variations (Wong et al, 2011), are merged. For variants that could not be found in the SNVBox database, the feature vectors were filled with zeros. Before concatenation, each feature vector passes through a separate FC layer and sigmoid activation to address the scale difference between the two different input features. Then the concatenated features and the final FC layer is used to decide pathogenicity of a variant by applying softmax activation to classify the variant as pathogenic or benign. The binary cross entropy between labels and the predicted classes becomes the loss function of the network.

**4. Optimizing the pathogenicity predictor using training data**

  We trained the model with two steps. First, we used general conservation data to train the deep neural network mentioned above. As the network is trained with a huge number of sequences along with their evolutionary history and structural traits, the features of the networks are optimized to interpret the impact of each variant in various aspects. Among those features in the network, the concatenated features between extracted features representing sequence information and SNVBox features are used as the features to train further machine learning methods.

  Then, based on the features from the deep neural networks, we trained the variants from ClinVar database, common variants from GnomAD database, and the simulated variants we generated for STK11 protein. We made use of the Xgboost algorithm (Chen, Tianqi, and Carlos Guestrin, 2016) to train these variants. We applied two different sets of training data to build different models: 1) merged data of ClinVar variants and STK11 conservation variants. 2) merged data of ClinVar variant, common variants, and STK11 conservation variants. Scores for the “prediction” column are in the continuous closed interval [0, 1], where scores closer to 0 are predicted more likely to be no activity. The model network from section 4 is already trained on the contextual change driven by mutations. As such, we obtained the score for the synonymous mutation case at each position for the STK11 protein, then averaged them across all positions to establish a simulated baseline (wildtype) level of activity. We then scaled all scores using this baseline to obtain scores in the “activity” column.

**5. Structure-based Analysis**

As an alternative method, the structural impact and physicochemical property change of each variant was assessed based on the structure given by AlphaFold (Jumper et al. 2021, ID: AF-Q15831-F1). For the structural impact, five criteria, which include “on the secondary structure”, “side-chain collision”, “removal of polar interaction”, “located at internal region”, “located near active sites”, were quantitatively evaluated ranging from 0 to 1. The active sites were curated based on disease-causing variants reported at UniProt (The UniProt Consortium, 2021). Physicochemical property changes were evaluated based on additional four criteria including “hydrophobicity change”, “polarity change”, “size increment by 3 atoms'', “involvement of special amino-acids (Cys, Sec, Gly, Pro)”. Then, those scores, measured by nine criteria in total, were normalized based on five different significance weights for each criteria.

**6. Molecular Dynamics simulation**

We utilized Gromacs (version 2021.3) Molecular Dynamics simulations to evaluate the

structural impact of each variant. The wild-type structure was given by AlphaFold (Jumper et al. 2021, ID: AF-Q15831-F1), while the structure model for each variant was built by FoldX. For the deletion variant (K84_84del), MODELLER was used to build the model. Each structure model was simulated with the time step of 2 fs until the total step reached 5,000,000. Therefore, the overall simulation time was 10 ns. We divided the simulation time into 4 different sections, which were 2~4 ns, 4~6 ns, 6~8 ns, and 8~10 ns. For each time section, the average Root Mean Square Distance was measured for variants and compared with that of the wild-type structure. predicted activity of STK11 structures were then estimated based on the RMSD ratio between variants and wild-type.

**Prediction results**

**3billion_modelnumber_1.tsv**

: XgBoost model trained by ClinVar variants and grouped STK11 conservation variants

**3billion_modelnumber_2.tsv**

: XgBoost model trained by ClinVar variants, common variants with AF ≥ 0.1% and grouped STK11 conservation variants

**3billion_modelnumber_3.tsv**

: XgBoost model trained by ClinVar variants

**3billion_modelnumber_4.tsv**

: XgBoost model trained by ClinVar variants and common variants with AF ≥ 0.1%

**3billion_modelnumber_5.tsv**

: structure-based analysis of each variant based on the structure given by AlphaFold

**3billion_modelnumber_6.tsv**

: predictions generated using simulation of molecular dynamics (Gromacs)

**References**

Adzhubei,I.A. *et al.* (2010) A method and server for predicting damaging missense mutations. *Nat. Methods*, **7**, 248–249.

Chen, Tianqi, and Carlos Guestrin. (2016) Xgboost: A scalable tree boosting system. *Proceedings of the 22nd acm sigkdd international conference on knowledge discovery and data mining*.

Church,D.M. *et al*. (2011) Modernizing reference genome assemblies. *PLoS Biol.*, **9**, e1001091.

den Dunnen,J.T. *et al.* (2016) HGVS Recommendations for the Description of Sequence Variants: 2016 Update. *Hum. Mutat.*, **37**, 564–569.

Gilissen,C. *et al.* (2012) Disease gene identification strategies for exome sequencing. *Eur. J. Hum. Genet.*, **20**, 490–497.

Hochreiter,S. and Schmidhuber,J. (1997) Long Short-Term Memory. *Neural Comput.*, **9**, 1735–1780.

Jumper,J. *et al.* (2021) Highly accurate protein structure prediction with AlphaFold. *Nature* **596**, 583–589.

Karczewski,K.J. *et al.* (2020) The mutational constraint spectrum quantified from variation in 141,456 humans. *Nature* **581**, 434–443.

Pruitt,K.D. *et al.* (2005) NCBI Reference Sequence (RefSeq): a curated non-redundant sequence database of  genomes, transcripts and proteins. *Nucleic Acids Res.*, **33**, D501-4.

Remmert,M. *et al.* (2012) HHblits: Lightning-fast iterative protein sequence searching by HMM-HMM alignment. *Nat. Methods*, **9**, 173–175.

Rentzsch,P. *et al.* (2019) CADD: predicting the deleteriousness of variants throughout the human genome. *Nucleic Acids Res.*, **47**, D886–D894.

Ruder,S. (2017) An Overview of Multi-Task Learning in Deep Neural Networks.

Shihab,H.A. *et al.* (2013) Predicting the Functional, Molecular, and Phenotypic Consequences of Amino Acid Substitutions using Hidden Markov Models. *Hum. Mutat.*, **34**, 57–65.

Suzek,B.E. *et al.* (2007) UniRef: comprehensive and non-redundant UniProt reference clusters. *Bioinformatics*, **23**, 1282–1288.

The UniProt Consortium (2021) UniProt: the universal protein knowledgebase in 2021

*Nucleic Acids Res.* **49:D1**, D480-D489.

Wong,W.C. et al. (2011) CHASM and SNVBox: toolkit for detecting biologically important single nucleotide mutations in cancer. *Bioinformatics*, **27**, 2147–2148.

Won,D. et al. (2021) 3Cnet: pathogenicity prediction of human variants using multitask learning with evolutionary constraints. *Bioinformatics,* **btab529***,* 1-9.
